# Novel Insights into the Enrichment Pattern of Aroma and Taste in Cooked Marinated Meat Products of Black Pork via Typical Process Steps

**DOI:** 10.3390/foods13223643

**Published:** 2024-11-15

**Authors:** Haitang Wang, Jiapeng Li, Yan Zhao, Qiang Li, Shouwei Wang

**Affiliations:** 1 China Meat Research Centre, Beijing 100068, China; haitang0414@163.com (H.W.); ljp7915@126.com (J.L.); cmrczy@126.com (Y.Z.); 2 Beijing Academy of Food Sciences, Beijing 100068, China; 3 College of Science, Beijing Forestry University, Beijing 100091, China; liqiang@bjfu.edu.cn

**Keywords:** cooked marinated pork knuckle, odour-active compounds, taste-active compounds, evolution, insights

## Abstract

This study aims to reveal the evolution mechanism of odour and taste active compounds in cooked marinated pork knuckles via typical process steps; among them, the brine soup stage was the most important part due to spices’ enriching flavours. These results revealed that the content and diversity of volatile compounds increased due to the addition of spices and heating temperature, imparting a unique aroma. Aldehydes played the main role in the overall odour. Benzaldehyde, hexanal, 1-octen-3-ol, levulinic acid, hydroxyacetone, ethyl octanoate, and 2-pentyl-furan were identified as the most important odour-active compounds. The key taste-active amino acids were glutamine, leucine, valine, and lysine. The IMP, AMP, and GMP provided a strong umami taste. Taste nucleotides and Val, Leu, Ile, and Phe were important precursor substances for aldehydes. The high responses of the electronic nose indicated that the gas component contained alkanes, alcohols, and aldehydes. The synergistic effects between umami-free amino acids and nucleotides correlated well with umami, as assessed by the electronic tongue. These results could be a starting point for the manufacturing industry, contributing to a better understanding of product performance.

## 1. Introduction

According to the OECD (2020), pork is a widely consumed meat worldwide and holds the position of the second most consumed meat product. It is noteworthy that China leads as the largest producer and consumer of pork on a global scale [1,2]. Consumers are drawn to Chinese black pork (CBP) due to its excellent nutritional content, tender texture, delicious taste, and unique flavour. Among the various CBP options, Beijing Heiliu (BH) pork stands out as a prime example within China’s local market [3]. The unique processing techniques of traditional cuisine include smoking, grilling, marinating, and boiling. Marinating and boiling are the most nutritious meat processing techniques. When the pork is marinated and stewed, some substances dissolve into the water, and these reaction substances participate in a series of biochemical reactions. That is, brine soup is gradually formed, and then the cooked marinated meat products are formed. As a traditional Chinese meat product, cooked marinated meat products have always been preferred by consumers because of their rich flavours [4]. There are many nutrients in brine soup, including free amino acids, B vitamins, small peptides, creatine, and other substances. They are easily absorbed and utilised by the human body. The degradation and interaction of these substances occur through a sequence of thermal reactions, including the Maillard reaction, lipid oxidation, thiamine’s thermal degradation, and the formation of volatile and nonvolatile compounds. These processes determined the odour properties and were responsible for most of the distinctive flavours found in meat products [5].

Flavour is not only one of the most prominent indicators for evaluating the quality of meat products, but also a great sensory property that affects the preferences of consumers [6]. For traditional meat cuisines, special production techniques inspire their unique flavours, which are also an embodiment of the Chinese food culture. Nevertheless, effectively replicating the authentic flavours of traditional cuisine through industrial production or intelligent and standardised processing remains a significant obstacle [7]. As a result, it is essential to thoroughly comprehend the evolution and formation route of unique odour and taste during traditional processing. This knowledge would offer a distinct direction for the refinement and standardisation of traditional pork dishes. Flavour comprises odour (volatile compounds) and taste (nonvolatile compounds). Odour-active compounds are responsible for the distinctive aroma qualities found in meat products and originate from the various chemical processes of precursor content in raw meat during heating and other processes [8]. It is indispensable for forming distinct taste attributes via nonvolatile compounds, including free amino acids and nucleotides [9]. Meanwhile, free amino acids and nucleotides can be involved in the Maillard reaction and Strecker degradation to form key precursors of flavours, which are important for the development of characteristic odours [10].

Over the past few years, there has been some research on the characterisation of lipids and volatile compounds or meat quality traits of black pork raw meat [11]. It is noteworthy that there is still a lack of reports on black pork cooked marinated meat products. For traditional cooked marinated meat products, most of these studies have concentrated on improving technology for processing meat products and adding flavour enhancers to increase their flavours. Wei et al. [12] enhanced the volatile compounds in Chinese marinated chicken through a combination of quantitative marinating and Maillard reaction. Few reports have investigated the evolution of taste and odour during the processing of actual dishes. Hence, it is of utmost importance to analyse odour and taste changes that occur during the actual heating process and understand the impact of traditional processing methods on the development of the characteristic meat flavour is of utmost importance.

Based on the above situation, the test was to reveal the odour and taste evolution and formation mechanisms of cooked marinated meat products throughout the production process. The content of volatile compounds, free amino acids, and nucleotides during the production process was measured. Additionally, the study explored changes in the type and content of key odour-active and taste-active compounds through multivariate statistics. Meanwhile, the electronic nose and electronic tongue were used to analyse the evolution of odour and taste characteristics, and the synergistic effects of amino acids and nucleotide on the evolution of taste properties. These findings provided a great understanding of traditional cuisine’s odour and taste evolution mechanism. It could be a starting point for the manufacturing industry, which make a contribution for understanding product performance and covering a transition from traditional methods to more industrialised and standardised processes.

## 2. Materials and Methods

### 2.1. Sample Preparations

Twenty-five Beijing Heiliu black pigs weighing approximately 90 kg at about 7 months were employed as the source of meat from Beijing Heiliu Animal Husbandry Technology Co., Ltd. (Heiliu, Beijing, China). The meat was obtained from the fore knuckle of different batches, 24 h after slaughter. All standard chemicals were obtained from Sigma (Agilent, Santa Clara, CA, USA). All other chemicals were of analytical reagent grade.

**Figure 1:**
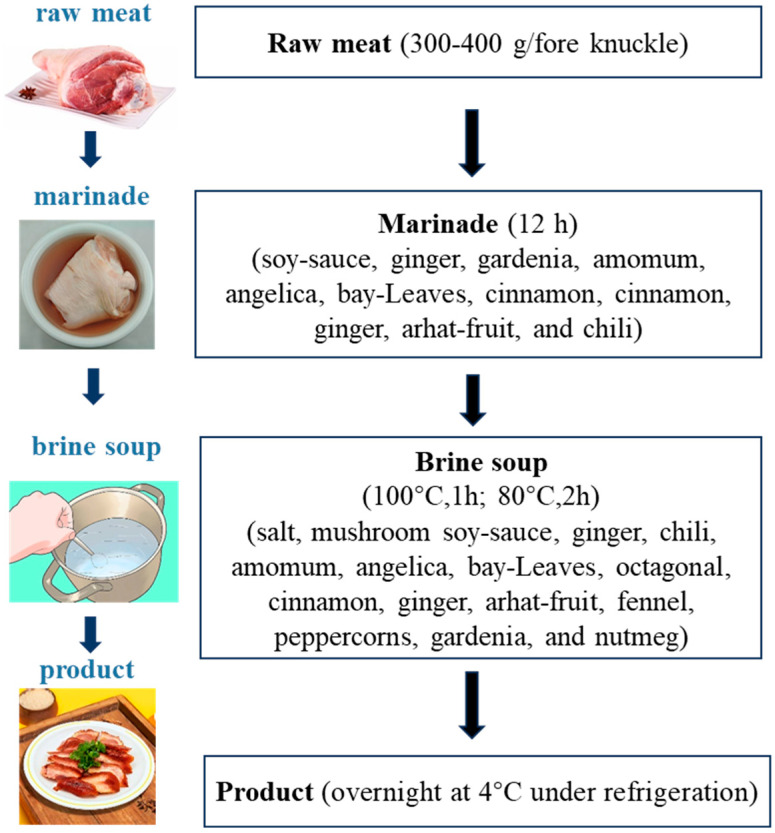
A manufacturing flowchart of cooked marinated pork knuckle.

Ten groups of fore knuckle samples (five samples from each group, 300–400 g for each sample). The complete preparation method of traditional cooked marinated pork knuckle was shown in Figure 1. Typical steps in the machining process were performed, which including the raw meat stage, marinade stage, simmering in brine soup stage, and finished product stage were collected as samples and were labeled as T1, T2, T3, and T4 (four treatments), respectively. The pork knuckle should be boneless, ensuring that the epidermis of the sample was intact during the process. The marinade consisted of raw meat, water, soy sauce, ginger, gardenia, amomum, angelica, bay leaves, octagonal, cinnamon, ginger, arhat fruit, and chili in a mass ratio of 100:50:9:0.1:0.01:0.01:0.01:0.01:0.015:0.015:0.01:0.015:0.03. The time of marinade was 12 h. The brine soup consisted of marinaded meat, water, salt, mushroom soy sauce, ginger, chili, amomum, angelica, bay leaves, octagonal, cinnamon, ginger, arhat fruit, fennel, peppercorns, gardenia, and nutmeg in a mass ratio of 100:200:1.05:1.75:0.1:0.525:0.01:0.0275:0.015:0.07:0.065:0.02:0.01:0.06:0.0525:0.01:0.01. The samples were boiled at 100 °C for 1 h and then soaked at 80 °C for 2 h and then overnight at 4 °C under refrigeration to obtain the finished product.

### 2.2. Analysis of Volatile Compounds

The types and contents of volatile compounds in the headspace vials were determined by using headspace solid-phase micro-extraction (HS-SPME) and gas chromatography/mass spectrometry (GC/MS-TSQ8000, Thermo Fisher Scientific Co., Ltd., Waltham, MA, USA), which was reported by Wang et al. [13] with some modification. An SPME fibre coated with 50/30 μm of divinylbenzene/carboxen/polydimethylsiloxane (DVB/CAR/PDMS) (Supelco, Bellefone, PA, USA) was used to extract the volatile compounds. In total, 5 g samples were put into a 20 mL clamp headspace vial (5183-4474, Agilent, CA, USA), and 1 μL of 2-methyl-3-heptanone was used as an internal standard (0.816 μg/μL). Volatile compounds in headspace vial were extracted at 50 °C in the bath for 30 min after equilibrating at 50 °C for 10 min, and the adsorbed compounds were desorbed in the GC injector for 6 min at 250 °C to avoid residue using the splitless mode. Volatile compounds were separated using a TG-Wax MS polarity column (30 m × 0.25 mm × 0.25 μm). High-purity helium (>99.99%) was used as the carrier gas with a column flow rate of 1.0 mL/min. The chromatograph oven temperature was held at 40 °C for 3 min, raised from 40 °C to 200 °C at 5 °C/min and maintained for 2 min, and subsequently raised from 200 °C to 230 °C at 10 °C/min and maintained for 3 min. The MS was operated in the full scanning mode over a range of *m*/*z* 40–600, and the scan time of 2 s. The voltage was set at 1.2 kV, the electron bombardment ion source temperature was 280 °C, and the electron ionisation was conducted at 70 eV.

### 2.3. E-Nose Detection

A portable PEN3 E-nose (Airsense Analytics GmbH, Schwerin, Germany) was used to record data. The sensor array consists of ten sensors, and the principle of operation is that odours are absorbed by the sensor array, resulting in a change in conductivity. The sample (1 g) was accurately weighed in the 7.5 mL headspace vial and sealed. When the sample collection is complete, clean air filtered through activated carbon will be pumped into the electronic nose again for the purpose of cleaning the sensor and restoring it to its original state. The purpose is to clean the sensor and bring it back to its original state by pumping clean air that has been passed through activated carbon into the electronic nose once more after the sample collection is completed. The temperature of the water bath was set at 50 °C, and the sample vials were put in it to balance 180 s, which is washing time of the electronic nose machine. The signal acquisition time was chosen to be in the 70 s, and data were collected.

### 2.4. E-Tongue Detection

The sourness, bitterness, saltness, umami, astringency, and richness of the sample were measured using an electronic tongue system. The E-tongue detection was conducted using the SA402B E-tongue (Ensoul Technology Ltd., Beijing, China) according to the method of Wu et al. [14] with minor modifications. An approximately 10 g sample was added with 100 mL of ultrapure water. The sample was homogenised and mixed for 60 s. These solutions were centrifuged at 8000× *g* for 5 min at 4 °C and filtered to remove solids with quantitative filter paper. A total of 60 mL liquid was collected for the test. The testing and washing times were 120 and 10 s, respectively.

### 2.5. Detection of Free Amino Acids

According to the method of Wu et al. [14] with some modification, sample (1 g) was mixed with 15 mL of 0.02 mol/L dilute hydrochloric acid and homogenised for 5 min. The solution was centrifuged at 8000× *g* for 10 min; then, the supernatant was put into the 25 mL volumetric bottle. Adding 1 mL of 5% sulfosalicylic acid (*v*/*v*) to 1 mL solution, and the mixed solution was centrifuged at 10,000× *g* for 10 min at 4 °C, and the clarified filtrate was filtrated by 0.22 μm water phase filter membrane. The supernatant was analysed using an Amino Acid Automatic Analyzer (L-8900, Hitachi, Tokyo, Japan). The separation column was an ODS Hypersil (250 mm × 4.6 mm × 5 μm), and the temperature was set at 40 °C. After filtration, the solution was pumped into a small glass vial, and the amount of liquid added was ensured to exceed the lowest white mark on the vial. The results are the average value of three parallel tests. Samples can be kept at 4 °C for refrigeration before detection.

### 2.6. Detection of Nucleotides

Inosine monophosphate (IMP), adenosine monophosphate (AMP), guanosine monophosphate (GMP), hypoxanthine (Hy), and inosine (In) constitute nucleotide compounds. These contents of nucleotide were measured following the method described by Zhan et al. [15] with some modifications. The sample (5 g) was accurately weighed, and then the sample homogenised with 25 mL of 5% precooled HClO_4_ for 2 min. At last, the solution was mixed at room temperature for 30 min before being centrifuged at 8000× *g* for 10 min at 4 °C. A filter paper was used to filter the supernatant, and the above condition was repeated. After adjusting the pH to 6.5–7.0 using 5 mol/L KOH, the solution was diluted to 100 mL using ultrapure water and filtered with a 0.45 μm filter membrane for subsequent detection using HPLC (Thermo Fisher Scientific Co., Ltd., Waltham, MA, USA).

### 2.7. Taste Activity Value (TAV) and Equivalent Umami Concentration (EUC)

The value of TAV is calculated as the ratio of the content of the taste compound to its threshold value. An equivalent umami concentration (EUC) was used to evaluate the umami intensity of the food. The synergistic actions of nucleotides (AMP, IMP, and GMP) and umami amino acids (glutamic acid and aspartic acid) are responsible for this achievement. Meanwhile, it was determined through the following equation:

$$\mathrm{EUC}=\sum {a_{i}b}_{i}+1218\left(\sum {a_{i}b}_{i}\right)(\sum {a_{j}b}_{j})$$

where the concentration (g/100 g) of umami amino acids (aspartic or glutamic acid) and umami nucleotides (IMP, GMP, or AMP) is represented by *a_i_* and *a_j_*, respectively. The relative umami concentration (RUC) for each umami amino acid to MSG (glutamic acid: 1 and aspartic acid: 0.077) and the RUC for each umami nucleotide to IMP (IMP: 1, GMP: 2.3, and AMP: 0.18) is represented by *b_i_* and *b_j_*, respectively. The constant 1218 represents a synergistic constant that is determined by the concentration of g/100 g employed.

### 2.8. Statistical Analysis

Three independent batches of samples (replicates) were prepared, and all measurements were conducted in triplicate (triplicate observations) for each batch. Data analysis was performed by using Statistix 8.1 software packages (Analytical Software, St Paul, MN, USA) and the results were expressed as the mean values ± standard deviation (SD). Significant differences between the means (*p* < 0.05) were confirmed by a one-way analysis of variance (ANOVA) with Tukey’s multiple comparisons.

## 3. Results and Discussion

### 3.1. Changes of Volatile Flavours in Cooked Marinated Pork Knuckle During Processing

There were 20 compounds in raw meat (T1), including 3 aldehydes, 2 ketones, 4 alcohols, 4 esters, 4 acids, and 3 alkenes in Table 1. It is worth noting that the content of 1-nonanol was the highest in the T1 stage, which was decreased in the marinade stage (T2), and even not detected in the brine soup stage (T3) and finished product stage (T4). It suggested that the 1-nonanol is from raw meat and is easily decomposed, which has a pleasant aroma of rose and orange. In addition, vinyl hexanoate is a sweet-smelling substance unique to black pork and has a well-rounded, pineapple-scented aroma. Methyl heptanone, with a fruity and fragrant flavour, has the effect of enhancing the aroma of meat, and it is mainly derived from raw meat. Compared with raw meat, the types and contents of volatile compounds increased significantly (*p* < 0.05) in the T2 stage, in which the pork knuckle underwent a marinade process using salt and spices [16,17]. There were 33 compounds measured in T2, including 5 aldehydes, 2 ketones, 8 alcohols, 6 esters, 4 acids, 1 furfural, 3 phenols, and 4 alkenes. Furfural and 2-furan methanol were not found in raw meat. This indicated that they are the characteristic flavour compounds of soy sauce.

There were 40 compounds measured in T3, including 9 aldehydes, 3 ketones, 10 alcohols, 6 esters, 5 acids, 3 phenols, 1 furan, 1 furfural, and 3 alkenes. There were 60 compounds measured in T4, including 15 aldehydes, 6 ketones, 14 alcohols, 8 esters, 9 acids, 3 phenols, 1 furan, 1 furfural, and 3 alkenes. The types and contents of flavour compounds increased significantly (*p* < 0.05) after heating. The degradation of protein, the Maillard reaction of flavour precursors, and the Strecker degradation of amino acids through heating are the factors that contributed to this result. In addition, spices play an important role in the processing of cooked marinated meat products, which can not only give meat products unique flavour and improve the flavour of meat products but also restrain and correct the unpleasant odour in meat products. The active ingredients in spices affect the formation of fat oxidation products. Meanwhile, spices can also react with raw meat components to produce volatile flavour substances. For example, some volatile compounds are from spices, and benzaldehyde has bitter almond, cherry, and nutty aromas derived from anise, cinnamon, and syzygium aromaticum; 4-Methoxy-benzaldehyde, which provides a long-lasting hawthorn aroma, derives from anise and verum; Linalool has floral, woody, berry and other aromas, and is from anise, verum, cinnamon, syzygium aromaticum, nutmeg, peppercorns and coriander seeds; α-Terpineol is from anise, verum, cinnamon, nutmeg, peppercorns, coriander seeds, and amomum tsao-ko; Eucalyptol is from verum, nutmeg, cinnamon, peppercorns, coriander seeds, and amomum tsao-ko; Terpinen-4-ol equipped with peppery is present in verum, nutmeg, cinnamon, peppercorns, and coriander seeds. And 4-Terpineol is from syzygium aromaticum. In particular, the content of anethole in T3 was high to 647.56 μg/g, which has a spicy and licorice smell mainly from anise, cinnamon and verum in the spices. Still its content declined significantly (*p* < 0.05) in the T4 stage, which indicates that it is easily soluble in water and easy to be enriched in the brine soup. In addition, the components of anise and verum would affect the fat oxidation products such as hexanal and octanal in raw meat [18].

**Figure 2:**
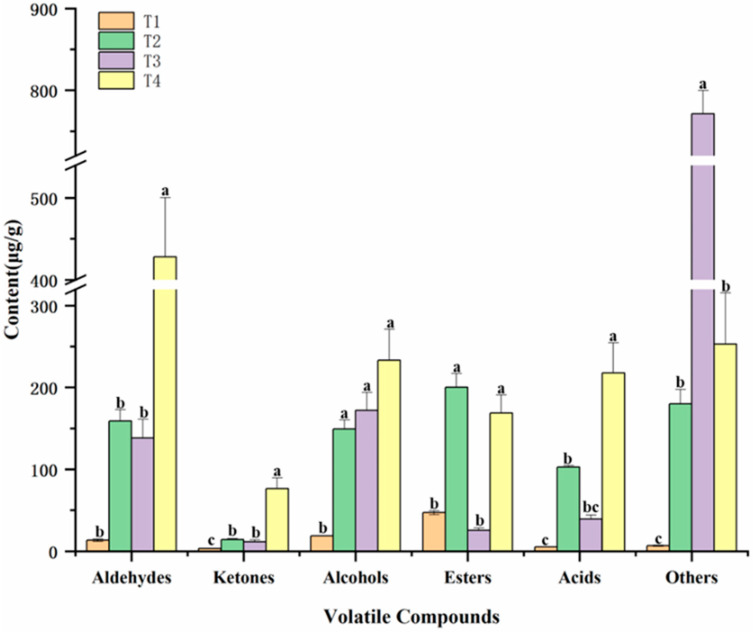
The changes in different groups of volatile compounds in cooked marinated pork knuckle during processing. The significant differences among different treatments are indicated by different uppercase letters (a–c) (*p* < 0.05).

Figure 2 displays the contents of different groups of volatile compounds. Aldehydes have a significant impact on the overall flavour of meat production due to their low threshold values [19]. Moreover, some short-chain fatty aldehydes (C5–C9) derive from the rapid oxidation of polyunsaturated fatty acids in lipids, and these changes are also critical to the overall flavours of cooked marinated pork knuckle. For instance, hexanal (OT = 5 μg/kg) has tallow, grass, and fat odours; heptanal (OT = 3 μg/kg) has sweet apricot, nutty aroma; octanal (OT = 0.7 μg/kg) has a strong fruity aroma; and nonanal (OT = 1 μg/kg) has fat and sweet orange notes. Benzaldehyde, which exhibits pleasant almond and burnt sugar odours (OT = 350 μg/kg), had higher (*p* < 0.05) contents in T_4_ than the other treatment groups and is generated from the degradation of phenylalanine [20]. In addition, trans-2-octanal has a fatty and meaty aroma; cis-2-nonenal has a roasted pork aroma. These aldehydes from lipid oxidation attend in the Maillard reaction during the heating stages. Ketones have little contribution to the overall flavour of cooked marinated pork knuckle due to lower concentration and higher threshold, and hydroxyacetone was from the Maillard reaction.

Alcohols accounted for the majority of the total volatile flavour compounds in this study, which originate from fat oxidation. Saturated alcohol has a high threshold and therefore has little effect on flavour. However, unsaturated alcohol has a lower threshold, can be easily perceived by humans, and makes a primary contribution to the overall flavour of cooked marinated pork knuckle [21]. The increasing content of 1-octen-3-ol indicated an increase in lipid oxidation, and oxidation resulting from thermal processing was a key cause of flavour production in meat, and it has a similar aroma to mushrooms and is a delicious soup flavour. In addition, according to the report, linalool exhibits bioactive properties, including antibacterial and sedative effects, along with the ability to reduce blood pressure [22]. Esters impart floral and fruity aromas to meat products with low odour threshold values and have a significant impact on flavour. The main esters detected in the samples were octyl octanoate with a brandy aroma, ethyl decanoate with a coconut aroma and ethyl hexanoate with a pineapple aroma. Short-chain acids (C < 6) contribute to the development of special flavour characteristics due to their low threshold values [23]. Medium-chain acids (C6–C12), such as octanoic acid and nonanoic acid, are from the degradation of lipids. They can act as precursors of flavour compounds, which indirectly influence flavour development. Typical products resulting from the degradation and reaction of free amino acids and proteins under thermal treatment included olefins, furans, furfural, and phenols, among other flavour compounds. Furthermore, hydrocarbons typically have a high threshold, and their effect on the overall flavour was not considerable. Among them, 2-pentyl-furan is a product of linoleic acid oxidation and has a low odour threshold (6 μg/kg). In addition, hydrocarbons typically have a high threshold, and their effect on the overall flavour was not considerable.

### 3.2. Analysis of Odour-Active Compounds in Cooked Marinated Pork Knuckle

**Figure 3:**
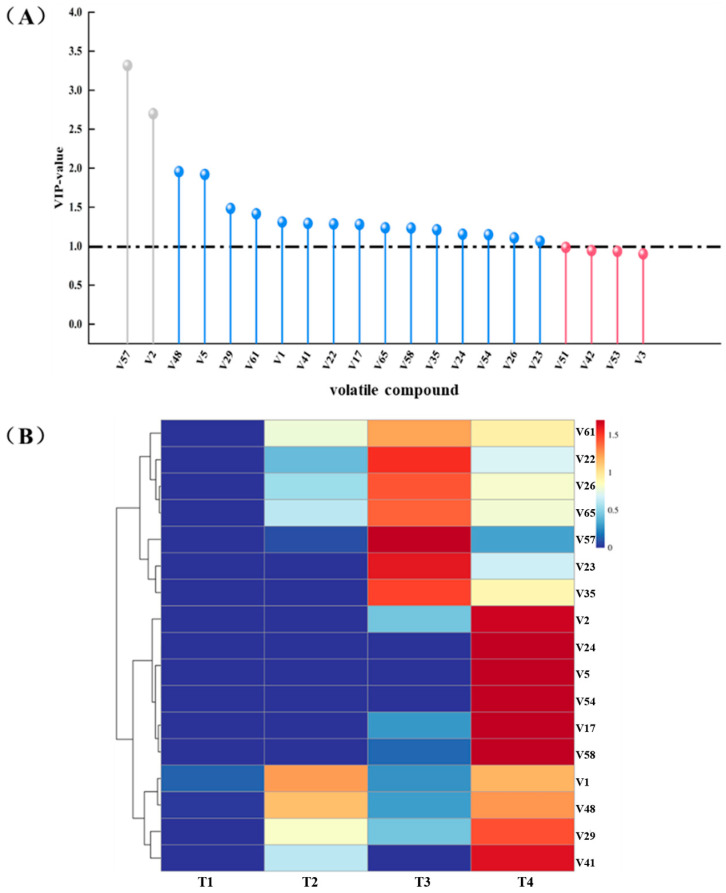
Variable importance projection (VIP) scores (**A**) and the heat map of volatile compounds (**B**) in cooked marinated pork knuckle during processing.

A variable importance for the projection plots (VIP) is used to evaluate the characteristic flavour of cooked marinated pork knuckle, in which a VIP > 1 indicates the volatile has crucial contributions to the overall flavour [24]. Partial least square discriminant analysis (PLS-DA) model was performed to predict the VIP values, and the Q^2^ and R^2^ of the PLS-DA models were 0.918 and 0.997, suggesting the models fitted well with the data in this study. As shown in Figure 3A, 17 key volatile compounds, including 3 aldehydes, 1 ketone, 6 alcohols, 1 ester, 2 acids, 2 phenols, 1 furan, and 1 alkene, were presented. In order to mirror the evolution and the spread of volatile compounds during processing, the content of volatile compounds was normalised, and a heat map was plotted as shown in Figure 3B. From the results, the T1 stage contained fewer compounds but high relative content, which may be the reason that the raw meat emitted a disagreeable smell. Although there was a slight increase in the light-yellow area of the compounds in the T2 stage, the majority of the compounds had a very low relative content. Most of the compounds in the T3 and T4 stages gradually turned a red colour, which indicated that both the content and the type of the volatile compounds in the cooked marinated pork knuckle were rising, resulting giving it a richer aroma. Through a combination of the VIP values and contents in heat maps, it can be supposed that benzaldehyde, hexanal, 1-octen-3-ol, levulinic acid, hydroxyacetone, ethyl octanoate, and 2-pentyl-furan were the most prevalent odour-active compounds of cooked marinated pork knuckle.

### 3.3. Changes in Taste Compounds in Cooked Marinated Pork Knuckle During Processing

**Figure 4:**
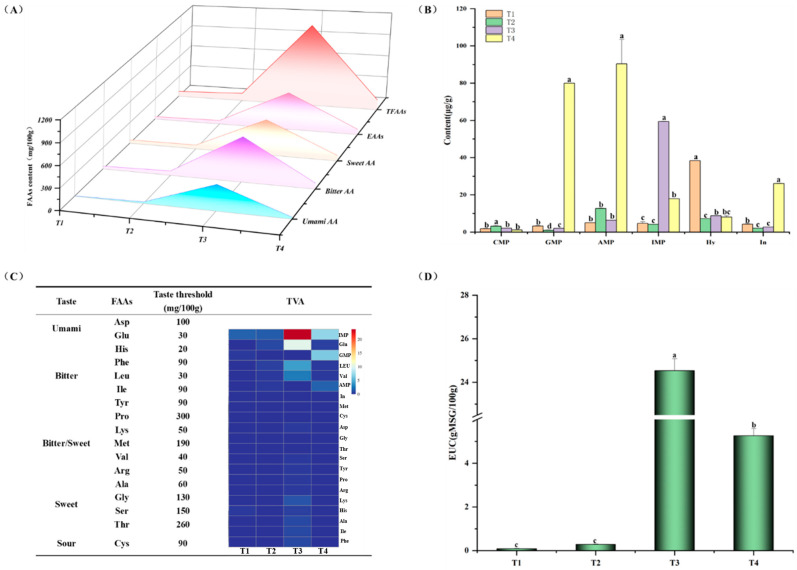
Changes in taste-active amino acids (**A**), nucleotides contents (**B**), TAV of amino acids (**C**), and EUC of nucleotides (**D**) in cooked marinated pork knuckle during processing. The significant differences among different treatments are indicated by different uppercase letters (a–d) (*p* < 0.05).

There are 17 free amino acids in Table 2, including 7 essential amino acids. The major free amino acids are glutamic acid, leucine, valine, proline, alanine and phenylalanine, and the T4 stage contained approximately 61% of the total free amino acids in its composition, which involved three essential amino acids. It has been reported that the content of Lys, Glu and Thr in black pork is higher than that of ordinary pork, and these three amino acids play an important role in the formation of flavour [3]. The content of amino acids rises with the progress of protein hydrolysis. Among them, the concentration of Glu, which is a typical amino acid that contributes to umami taste, was relatively high. Furthermore, the amount of Glu consistently increased as the heating process continued. Glu and Asp have an acidic taste; when sodium salts are present, they provide a strong umami taste [6]. Moreover, the contents of sweet amino acids Ala and Thr were relatively high in the T1 stage. From Figure 4A, all free amino acid contents were high in the T3 stage, and the rich taste of the sample was provided by brine soup. Bitter and sweet amino acids are the most abundant in the brine soup, followed by umami amino acids, and astringent amino acids are the least abundant due to their unfavourable contribution to the taste. The desirable taste of cooked marinated pork knuckle could be attributed to these factors. During the processing, free amino acids participated in the Maillard reaction and form different flavour precursors, which are added to the flavours of meat products. Similar findings reported by Rotola-Pukkila et al. [25] found that hydrolysis of proteins and peptides into amino acids due to thermal treatment, and the free amino acids would be released into pork cooking juice. For example, Met is a dominant precursor substance for sulphur-containing volatile compounds, which could contribute to the development of meaty and soy-like flavours. In addition to their sweet characteristics, Ala, Gly, Ser, and Thr can undergo Streker degradation to become aldehydes, thereby stimulating the production of highly aromatic pyrazines. Val, Leu, Ile, and Phe are degraded by Streker to form branched chain aldehydes and aromatic aldehydes.

The essential taste-active compounds in meat production involve amino acids and nucleotides. Flavour nucleotides have a mild salty taste and possess broth-like and meaty notes. The content of nucleotides is listed in Figure 4B. It is a great contribution of nucleotides to the taste in meat, in which the synergistic effect of nucleotides with Glu and Asp significantly raises the umami perception [26]. During heating, the contents of umami nucleotides were increased, especially in IMP, AMP, and GMP, and this factor could contribute to the enhanced flavour experienced in cooked marinated pork knuckle after it has been cooked. Meanwhile, the IMP degraded into In due to the thermal reaction, which could endow meat with better taste. An increased concentration of Hy could result in a bitter taste [27]. After being heated, the Hy content decreased significantly (*p* < 0.05) and remained lower than in the raw meat (T1 stage). This decrease played a role in reducing the bitterness in the cooked marinated pork knuckle. Taste nucleotides, including GMP, IMP, and AMP, make a major contribution to meat taste perception. The content of AMP, GMP, and AMP was relatively high in the T4 stage. Furthermore, the process of producing volatile compounds included utilising taste nucleotides as precursors of flavour in various reactions.

### 3.4. Evolution of Key Taste-Active Compounds in Cooked Marinated Pork Knuckle During Processing

Amino acid taste intensity values (TAV) can be used for nutritional evaluation of protein amino acids. The development of the most essential taste-active compounds in cooked marinated pork knuckle could be revealed using the TAV; the larger the TVA value, the greater the contribution. Taste-active compounds corresponding to TAV values greater than 1 are the main contributors to taste attributes. According to Figure 4C, the taste-active amino acids, including Glu, Leu, Val, and Lys, had TAVs greater than 1, suggesting that these amino acids were the primary contributors to taste-active compounds. Although the TAV values of remaining free amino acids were lower than 1, they still make some contribution in two ways: firstly, FAAs acted as thermal reactive reactants to produce flavour precursors, and secondly, FAAs had synergistic effects with other taste-active compounds to enhance taste quality. The TAV values of GMP, AMP and IMP were greater than 1. Furthermore, the combination of IMP with sweet-free amino acids, including serine, glycine, and alanine, resulted in a synergistic effect, greatly enhancing the overall taste quality [28]. The taste experience of food can be assessed by measuring the equivalent umami concentration (EUC), which determines the effect of amino acids (ASP and Glu) and nucleotides (GMP, AMP, IMP) on taste intensity, comparing it to umami [29]. Figure 4D showed EUC changes during production process. EUC increased with the progress of heating. In addition, the findings from EUC and TAV results validated the synergistic effects between these taste-active compounds. Especially in the T3 stage, it indicated that it contributed to umami improvement.

### 3.5. Evolution of Odour and Taste Properties Analysed Through Electronic Nose and Electronic Tongue

**Figure 5:**
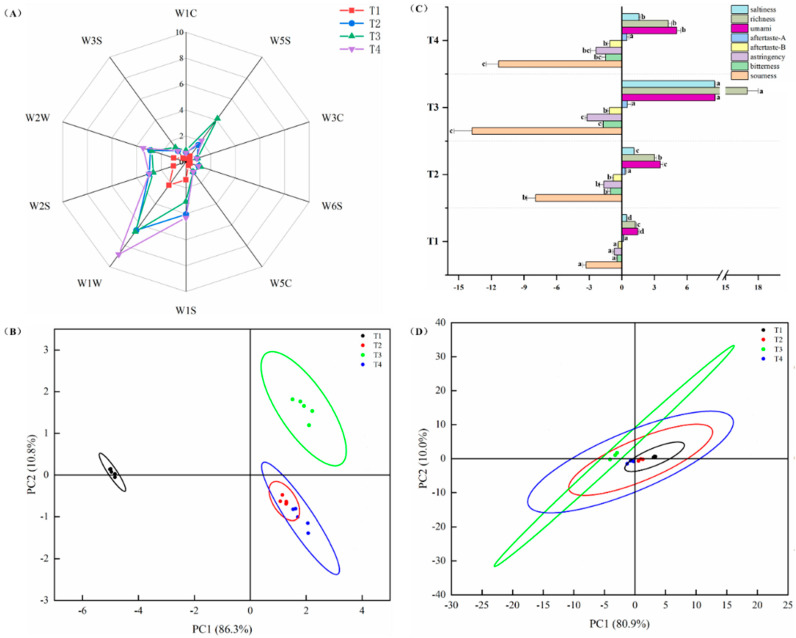
The sensor responses of the odour attributes of electronic nose (**A**), PCA analysis of electronic nose (**B**), the taste attributes of the electronic tongue (**C**), and PCA analysis of electronic tongue (**D**). The significant differences among different treatments are indicated by different uppercase letters (a–d) (*p* < 0.05).

The electronic nose can simulate the human olfactory system to achieve the quality assessment with simple sample pre-treatment, short detection time, easy-to-repeat, reliable results and so on [30]. Figure 5A displays the response of 10 electronic nose sensors to the odour of cooked marinated pork knuckle via processing. In T2, T3, and T4, the W2W, W2S, and W1S responses were approximately 3, suggesting the presence of alkanes, alcohols, aldehydes, and esters in the gas component. The findings were aligned with the GC/MS analysis, where the inclusion of spices amplified the odour of the cooked marinated pork knuckle during the heating stage. Figure 5B presents the PCA results of the electronic nose in cooked marinated pork knuckle. The PC1 and PC2 account for 97.1% of the total variance in the system (86.3% and 10.8%, respectively). The changes in the overall odour profile of the cooked marinated pork knuckle through processing were characterised by the PCA model. Meanwhile, the result of PC1 donated more effect on interpreting the information of the sample. Based on these, it is possible to identify the variations among samples based on their relative positions on the abscissa. The large horizontal distance between T1 and the other samples suggested that the odour profile changed as the temperature rose.

**Figure 6:**
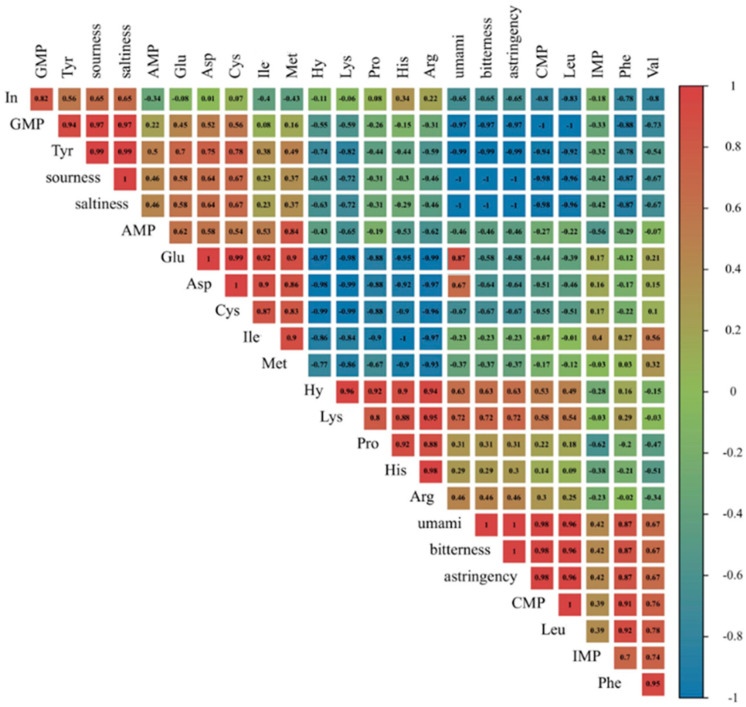
Heat map of the Pearson correlation between taste-active compounds and E-tongue.

The electronic tongue is also known as artificial taste recognition or taste sensor technology, and the purpose of the study was to assess the variations in the fundamental taste characteristics (umami, saltiness, astringency, bitter, and sour), which are shown in Figure 5C. There were some studies published the synergistic effects between umami-free amino acids; meanwhile, the correlation between umami detected via the electronic tongue and umami nucleotides based on EUC was strong [31]. These findings were also consistent with our study; that is, the value of EUC rose significantly (*p* < 0.05) with temperature, and the umami quality of cooked marinated pork knuckle was consistently enhanced while being heated. Furthermore, the electronic tongue detected the richness as the lingering taste of the umami substance, and so the richness was constantly increased during heating. Additionally, the concentration of sodium chloride could be intensified due to the heightened water loss, thus improving the saltiness in cooked marinated pork knuckle. As shown in Figure 5D, the saltiness increased due to the accumulation of umami. The reason for this is that umami compounds also exhibited properties of saltiness. The principal components accounted for 90.9% of the total variance, the PC1 component contributed 80.3%, and the PC2 component contributed 10.0%. The taste profile of samples was presented by PC1 and PC2 components. These findings displayed that the flavour quality of cooked marinated pork knuckle became consistent over the various stages. Figure 6 displays the Pearson correlation between taste-active compounds and sensory evaluation. For nucleotides, there was a positive correlation among CMP, IMP, and Hy with umami discovered by the E-tongue; the correlative values were *p* = 0.98, *p* = 0.42, and *p* = 0.63, respectively. However, the In (*p* = −0.65) and AMP (*p* = −0.46) were negatively relevant to umami. Furthermore, the free amino acids presented umami taste, including Asp (*p* = 0.67) and Glu (*p* = 0.87), were positively associated with umami. Meanwhile the Asp (*p* = 0.64) and Glu (*p* = 0.58) were positively associated with saltiness. The positive correlation between bitter taste FAAs, including His (*p* = 0.29), Leu (*p* = 0.96), Phe (*p* = 0.87), Arg (*p* = 0.46), Val (*p* = 0.67), Lys (*p* = 0.72), and bitterness discovered by the E-tongue. The Cys (*p* = 0.67) was positively correlated with sourness.

## 4. Conclusions

The brine soup stage was the most important part due to the enriched variety of flavours provided by spices. Aldehydes played a vital position in the overall flavour. Benzaldehyde, hexanal, 1-octen-3-ol, levulinic acid, hydroxyacetone, ethyl octanoate, and 2-pentyl-furan were identified as the most predominant odour-active compounds. Glutamine, leucine, valine, and lysine were the significant taste-active amino acids. The IMP, AMP, and GMP provided a strong umami taste. In addition, taste nucleotides and Val, Leu, Ile, and Phe were important precursor substances for aldehydes. Furthermore, not only did the accumulation of free amino acids and nucleotides provide the substrate for thermal reactions, but there was also a synergistic effect between them, which contributed to the desired taste. The high responses of the electronic nose indicated that the alkanes, alcohols, and aldehydes existed in the gas component. The electronic tongue showed a strong correlation between the umami taste evaluated and the combined impact of umami-free amino acids and umami nucleotides when considering their equivalent umami concentration. These results could be a starting point for the manufacturing industry. Furthermore, they also aid in the standardisation and industrial production of traditionally processed.

## Figures and Tables

**Table 1:** Volatile compounds identified and quantified (μg/g) by gas chromatography/mass spectrometry in cooked marinated pork knuckle during processing.

Number	Compound	RI Value	T1	T2	T3	T4
	Aldehydes					
V1	Nonanal	1114	7.38 ± 1.23 ^b^	67.92 ± 6.34 ^a^	14.42 ± 1.74 ^b^	64.07 ± 22.68 ^a^
V2	Benzaldehyde	1227	n.d.	n.d.	38.48 ± 14.15 ^b^	146.15 ± 20.37 ^a^
V3	Octanal	994	n.d.	7.72 ± 0.56 ^b^	5.82 ± 0.24 ^b^	13.66 ± 1.66 ^a^
V4	Trans-2-Octanal	1125	n.d.	n.d.	4.52 ± 0.87 ^a^	4.10 ± 1.76 ^a^
V5	Hexanal	71	n.d.	n.d.	n.d.	99.03 ± 10.92
V6	4-methoxy-benzaldehyde	1614	n.d.	n.d.	17.54 ± 1.08 ^a^	9.12 ± 2.37 ^b^
V7	Cis-2-nonenal	1212	n.d.	n.d.	1.32 ± 0.26 ^b^	5.88 ± 0.55 ^a^
V8	Benzeneacetaldehyde		n.d.	54.08 ± 4.36 ^a^	15.92 ± 1.92 ^b^	9.63 ± 1.01 ^c^
V9	Hexadecanal	1636	4.65 ± 0.22 ^b^	4.79 ± 0.12 ^b^	5.64 ± 0.28 ^b^	8.30 ± 0.98 ^a^
V10	Heptanal	55	n.d.	n.d.	n.d.	14.99 ± 2.63
V11	2-Undecenal	1371	n.d.	n.d.	n.d.	3.25 ± 0.69
V12	Dodecanal	1491	1.45 ± 0.10 ^b^	5.01 ± 0.21 ^a^	n.d.	6.52 ± 1.43
V13	Octadecanal		n.d.	n.d.	n.d.	15.23 ± 1.11
V14	Decanal	1183	n.d.	n.d.	n.d.	12.64 ± 1.53
	Ketones					
V15	Methyl heptenone		0.88 ± 0.01 ^b^	0.92 ± 0.02 ^b^	1.88 ± 0.22 ^b^	10.17 ± 3.47 ^a^
V16	Hydroxyacetone	1008	n.d.	n.d.	5.91 ± 1.40 ^c^	36.29 ± 3.63 ^a^
V17	1-(Tetrahydrofuran-2-yl) ethanone		n.d.	n.d.	3.90 ± 0.34 ^b^	8.12 ± 2.72 ^a^
V18	3-hydroxy-2-butanone	998	2.25 ± 0.09 ^c^	13.31 ± 1.22 ^a^	n.d.	9.23 ± 1.19 ^b^
V19	2-Pentadecanone	1559	n.d.	n.d.	n.d.	8.24 ± 1.67
V20	2-Nonanone	1091	n.d.	n.d.	n.d.	4.29 ± 0.36
	Alcohols					
V21	Eucalyptol		n.d.	12.21 ± 0.75 ^b^	45.76 ± 5.24 ^a^	20.92 ± 5.40 ^b^
V22	Linalool		n.d.	n.d.	36.40 ± 4.40 ^a^	15.12 ± 0.04 ^b^
V23	1-Octen-3-ol	1146	n.d.	n.d.	n.d.	35.80 ± 8.70
V24	Phenylethyl Alcohol		n.d.	26.47 ± 2.65 ^a^	1.68 ± 0.08 ^c^	16.09 ± 2.80 ^b^
V25	α-Terpineol	1346	n.d.	10.06 ± 0.69 ^b^	26.63 ± 2.55 ^a^	15.36 ± 2.30 ^b^
V26	Undecanol	1525	2.33 ± 0.06 ^b^	5.23 ± 0.84 ^ab^	7.59 ± 1.75 ^a^	8.57 ± 0.33 ^a^
V27	5-methyl-2-Furanmethanol		n.d.	n.d.	5.35 ± 1.10 ^b^	10.64 ± 1.03 ^a^
V28	2-Furanmethanol		n.d.	24.67 ± 1.10 ^ab^	12.80 ± 1.56 ^b^	42.51 ± 12.42 ^a^
V29	1-Hexanol	1060	1.00 ± 0.01 ^b^	n.d.	n.d.	9.41 ± 1.71 ^a^
V30	1-Hexadecanol		n.d.	6.09 ± 0.65 ^b^	1.87 ± 0.61 ^c^	10.74 ± 0.29 ^a^
V31	2-Phenoxyethanol		2.43 ± 0.03 ^b^	58.51 ± 4.36 ^a^	n.d.	6.60 ± 1.89 ^b^
V32	Trans-2-octen-1-ol		n.d.	n.d.	n.d.	5.40 ± 0.30
V33	Trans 9-hexadecane-1-ol		n.d.	n.d.	0.98 ± 0.03 ^b^	15.87 ± 0.47 ^a^
V34	(-)-4-Terpineol		n.d.	n.d.	32.96 ± 4.45 ^a^	20.10 ± 0.16 ^b^
V35	1-Nonanol	1309	12.88 ± 0.11 ^a^	5.83 ± 0.30 ^b^	n.d.	n.d.
	Esters					
V36	Vinyl hexanoate		20.66 ± 0.67 ^b^	10.34 ± 1.21 ^c^	2.50 ± 0.22 ^d^	37.57 ± 1.34 ^a^
V37	12,15-Octadecylic acid methyl ester	35	n.d.	n.d.	1.30 ± 0.10 ^a^	2.77 ± 0.78 ^a^
V38	Isopropyl Palmitate		n.d.	n.d.	3.59 ± 1.92 ^b^	15.64 ± 1.16 ^a^
V39	Methyl cinnamate		n.d.	n.d.	15.17 ± 0.79	n.d.
V40	Ethyl octanoate		n.d.	17.21 ± 0.03 ^b^	n.d.	45.53 ± 16.67 ^a^
V41	Ethyl decanoate		n.d.	19.61 ± 01.5 ^b^	n.d.	27.87 ± 1.85 ^a^
V42	Isopropyl Myristate	1573	1.06 ± 0.01 ^d^	2.33 ± 0.04 ^b^	1.60 ± 0.08 ^c^	9.26 ± 0.11 ^a^
V43	Ethylene glycol laurate	1708	n.d.	n.d.	1.10 ± 0.05 ^b^	11.23 ± 0.17 ^a^
V44	Ethyl hexanoate		n.d.	n.d.	n.d.	18.63 ± 0.44
V45	Octyl octanoate		18.32 ± 0.67 ^b^	97.16 ± 11.67 ^a^	n.d.	n.d.
V46	2-Ethylhexyl ester		6.94 ± 0.98 ^b^	53.36 ± 2.67 ^a^	n.d.	n.d.
	Acids					
V47	Acetic acid	1154	2.70 ± 0.06 ^b^	91.75 ± 0.78 ^a^	24.00 ± 4.47 ^c^	101.43 ± 21.98 ^a^
V48	Octanoic Acid		0.73 ± 0.01 ^b^	5.32 ± 0.09 ^ab^	3.25 ± 0.02 ^b^	9.72 ± 2.94 ^a^
V49	Decanoic acid		0.63 ± 0.02 ^b^	2.85 ± 0.88 ^b^	1.01 ± 0.09 ^b^	13.00 ± 4.00 ^a^
V50	Nonanoic acid		1.03 ± 0.06 ^b^	2.83 ± 0.03 ^b^	n.d.	28.20 ± 1.77 ^a^
V51	Benzoic acid	1834	n.d.	n.d.	0.73 ± 0.21 ^b^	3.57 ± 0.40 ^a^
V52	Hexanoic acid		n.d.	n.d.	10.28 ± 0.23 ^b^	13.01 ± 0.22 ^a^
V53	Levulinic acid		n.d.	n.d.	n.d.	35.37 ± 4.53
V54	Butanoic acid	1228	n.d.	n.d.	n.d.	8.94 ± 0.05
V55	4-(hydroxyphenyl) phosphonic acid	1553	n.d.	n.d.	n.d.	4.59 ± 0.98
	Others					
V56	Anethole	1425	n.d.	31.12 ± 5.22 ^c^	647.56 ± 14.70 ^a^	119.30 ± 33.77 ^b^
V57	2-Pentyl-Furan		n.d.	n.d.	3.14 ± 0.32 ^b^	36.71 ± 9.09 ^a^
V58	Styrene	942	2.53 ± 0.60 ^b^	20.29 ± 0.20 ^a^	n.d.	19.79 ± 4.62 ^a^
V59	L-camphor		n.d.	56.22 ± 5.78 ^a^	13.42 ± 1.63 ^b^	24.19 ± 2.84 ^b^
V60	Dodecane	828	n.d.	21.06 ± 1.65 ^a^	33.22 ± 7.39 ^a^	25.35 ± 9.62 ^a^
V61	Pentyl-cyclopropane		n.d.	n.d.	n.d.	8.56 ± 0.90 ^a^
V62	Tetradecane	1011	0.46 ± 0.01 ^b^	5.76 ± 0.23 ^b^	40.23 ± 3.51 ^a^	n.d.
V63	Heptadecane		3.82 ± 0.11 ^b^	30.84 ± 4.21 ^a^	n.d.	n.d.
V64	Terpinen-4-ol		n.d.	14.64 ± 0.23 ^b^	33.62 ± 1.23 ^a^	19.17 ± 1.69 ^b^
V65	Furfural		n.d.	19.46 ± 2.36 ^a^	15.80 ± 0.79 ^a^	7.98 ± 0.64 ^b^
V66	5-methyl-furfural		n.d.	n.d.	18.99 ± 1.60 ^a^	7.47 ± 2.24 ^b^
Total			94.13 ± 5.06 ^c^	804.97 ± 62.88 ^ab^	1157.88 ± 82.87 ^a^	1376.89 ± 245.03 ^a^

n.d.: volatile compounds not detected. Different lowercase letters (a–d) in the same row indicate significant differences among different samples (*p* < 0.05). Treatments: T1: raw meat stage; T2: marinade stage; T3: simmer in brine soup stage; T4: finished product stage.

**Table 2:** Changes in free amino acid (FAA) of cooked marinated pork knuckle during processing.

Traits		Content (mg/100 g)			
		T1	T2	T3	T4
Umami taste FAAs	Asp	0.26 ± 0.01 ^c^	2.47 ± 0.01 ^b^	44.79 ± 0.91 ^a^	2.46 ± 0.30 ^b^
	Glu	5.03 ± 0.07 ^c^	24.37 ± 0.25 ^b^	301.73 ± 1.24 ^b^	19.53 ± 2.89 ^a^
Sweet taste FAAs	Ser	0.71 ± 0.25 ^c^	5.23 ± 0.08 ^b^	40.64 ± 0.67 ^a^	3.85 ± 0.53 ^b^
	Ala	11.30 ± 0.07 ^b^	10.60 ± 0.28 ^b^	50.08 ± 0.66 ^a^	8.92 ± 1.37 ^b^
	Gly	4.80 ± 0.06 ^b^	7.05 ± 0.24 ^b^	29.48 ± 1.20 ^a^	4.70 ± 0.66 ^b^
	Thr *	12.12 ± 0.54 ^b^	9.50 ± 0.09 ^c^	54.86 ± 0.12 ^a^	4.50 ± 0.68 ^d^
Bitter taste FAAs	His	6.12 ± 0.19 ^b^	2.07 ± 0.08 ^c^	10.89 ± 0.23 ^a^	1.08 ± 0.18 ^d^
	Ile *	2.30 ± 0.01 ^c^	8.50 ± 0.22 ^b^	74.92 ± 0.78 ^a^	6.83 ± 0.97 ^b^
	Leu *	5.29 ± 0.01 ^c^	15.78 ± 0.31 ^b^	124.64 ± 1.64 ^a^	12.10 ± 1.76 ^b^
	Phe *	3.59 ± 1.21 ^c^	10.02 ± 0.18 ^b^	65.82 ± 0.48 ^a^	8.36 ± 1.17 ^b^
	Tyr	0.99 ± 0.01 ^b^	0.07 ± 0.01 ^b^	23.13 ± 0.93 ^a^	0.16 ± 0.05 ^b^
Bitter/sweet taste FAAs	Pro	4.36 ± 0.05 ^c^	10.65 ± 0.42 ^b^	100.00 ± 1.38 ^a^	7.90 ± 0.70 ^b^
	Arg	4.04 ± 0.42 ^b^	3.62 ± 0.12 ^b^	14.10 ± 0.05 ^a^	3.06 ± 0.54 ^b^
	Val *	4.21 ± 0.07 ^c^	12.10 ± 0.16 ^b^	105.39 ± 0.31 ^a^	9.81 ± 1.39 ^b^
	Lys *	5.16 ± 1.10 ^c^	10.03 ± 0.17 ^b^	67.10 ± 0.59 ^a^	8.08 ± 1.17 ^bc^
	Met *	1.65 ± 0.01 ^c^	3.03 ± 0.20 ^b^	16.40 ± 0.16 ^a^	2.06 ± 0.09 ^c^
Sour taste FAA	Cys	0.68 ± 0.02 ^b^	0.81 ± 0.30 ^b^	11.58 ± 1.31 ^a^	1.05 ± 0.54 ^b^
Total FAAs		72.60 ± 4.08 ^c^	135.89 ± 3.10 ^b^	1135.54 ± 12.66 ^ba^	104.46 ± 15.07 ^bc^
EAAs		34.32 ± 2.95 ^c^	68.95 ± 1.33 ^b^	509.13 ± 4.08 ^a^	50.76 ± 8.65 ^bc^

Values in the same column with different letters were significantly different (*p* < 0.05). The mark * denotes essential amino acids (EAAs). Treatments: T1: raw meat stage; T2: marinade stage; T3: simmer in brine soup stage; T4: finished product stage.

## Data Availability

The original contributions presented in the study are included in the article, further inquiries can be directed to the corresponding author.
